# Supplementary material for: A Comprehensive Assessment of Ultraviolet-Radiation-Induced Mutations in Flammulina filiformis Using Whole-Genome Resequencing
Source: J Fungi (Basel). 2024 Mar 20;10(3):228. doi: 10.3390/jof10030228 (PMC10971301; doi:10.3390/jof10030228)
Supplement: Supplementary file 1 [file jof-10-00228-s001.zip › Supplementary Material S8/KEGG annotation/out/64381550635650.os/KO/out_map/map04151.html]

KEGG PATHWAY: PI3K-Akt signaling pathway - Reference pathway


|  |  |
| --- | --- |
| **PI3K-Akt signaling pathway - Reference pathway** |  |

[
Pathway menu
| Organism menu
| Pathway entry
| Show description
| User data mapping
]

The phosphatidylinositol 3' -kinase(PI3K)-Akt signaling pathway is activated by many types of cellular stimuli or toxic insults and regulates fundamental cellular functions such as transcription, translation, proliferation, growth, and survival. The binding of growth factors to their receptor tyrosine kinase (RTK) or G protein-coupled receptors (GPCR) stimulates class Ia and Ib PI3K isoforms, respectively. PI3K catalyzes the production of phosphatidylinositol-3,4,5-triphosphate (PIP3) at the cell membrane. PIP3 in turn serves as a second messenger that helps to activate Akt. Once active, Akt can control key cellular processes by phosphorylating substrates involved in apoptosis, protein synthesis, metabolism, and cell cycle.

|  |  |
| --- | --- |
| Reference pathway | 100% |
